# Analysis of barriers, supports and gender gap in the choice of STEM studies in secondary education

**DOI:** 10.1007/s10798-022-09776-9

**Published:** 2022-11-02

**Authors:** Noemí Merayo, Alba Ayuso

**Affiliations:** 1 Optical Communications Group of the Signal Theory, Communications and Telematic Engineering Department, E.T.S.I. Telecomunicación, Universidad de Valladolid, Paseo de Belén 15, Valladolid, Spain; 2 Department of Pedagogy, Faculty of Medicine, Universidad de Valladolid, Av. Ramón y Cajal, 7, 47005 Valladolid, Spain

**Keywords:** Technological vocations, Decline, STEM career interest, Gender gap, Secondary school students, Secondary school teachers

## Abstract

Society is more digitised than ever and there is an urgent need to train people in these sectors, where women are still under-represented. A quantitative descriptive, correlational and explanatory descriptive design was used to identify barriers, supports and gender gaps in Science, Technology, Engineering and Mathematics in Secondary Education by analysing the interest and perception of 1562 students and 432 teachers. Descriptive statistics, Chi-square and Lambda test and Crame’s V or Phi test were performed together with a qualitative analysis. The results show that fewer female students want to pursue STEM studies, with girls preferring health and education professions and boys preferring engineering and computer science. Indeed, their motivation is different since we found correlations between being a girl and choosing STEM for helping people and society, while earning money is important for boys. Girls believe more necessary than boys to have qualities to study STEM and less often perceive themselves as intelligent and courageous. Our study revealed that families and teachers encourage more boys than girls towards STEM activities. Teachers believe that girls are influence by preconceived ideas, lack of STEM knowledge and lower self-esteem. Regarding gender equality, almost half state that no objectives are included in the curricula, 43.85% do not include it in subjects and only 30% received training. Consequently, female vocations need to be promoted by teaching how STEM solves real-life problems, fostering creativity, increasing self-confidence, promoting STEM activities and making female role models visible. Teachers should receive more gender training and promote gender-sensitive STEM education.

## Introduction

The term STEM (Science, Technology, Engineering, and Mathematics) originated as a response to the growing demand for science and technology education due to constant technological change and an increasingly digitalised society. This term emerged in 2000 in the United States through the National Science Foundation and today it has become a relevant topic due to the urgency of closing the existing gender gap in these disciplines. Thus, gender gap is defined as the difference in attitudes, behaviours or skills, between men and women and was first used by the American feminist Eleanor Smeal in 1980. However, the minority representation of women in scientific and technological careers, which establishes the origin of the gender gap in STEM, dates back to the 1960s (Rossi, 1965). As the years progressed, authors such as Rees (2001) began to realise that women had moved from exclusion to segregation in the field of science, so that the representation of women continued to be in the minority compared to the majority of men who are trained and occupy scientific-technological professions. However, concern and sensitivity to the gender gap in science and technology has become more consolidated from the 1990s to the present day.

Current societies are becoming digitised at a frenetic and irreversible pace and the STEM sector represents an essential employment niche for the evolution of these societies, so these disciplines are present in all areas of life, including our relationships. Indeed, the Internet has been the lever of the digital transformation that all countries have undergone, accelerated by the pandemic and the health, economic and social crisis it has generated. This technological revolution requires professionals in areas such as Artificial Intelligence (AI), Big Data, Cybersecurity, Cloud Computing or Internet of Things (IoT) (Huawei, 2019). The European Commission (EU) claims that 45% of jobs in 2022 will be in the digital sector (fully related to all branches of STEM) with a highly increasing trend in the years to come (Rueda et al., 2021). In addition, STEM sectors are generating a huge demand for jobs, with more than 1.75 million jobs expected by 2030. By then, the most in-demand skills will be computer and programming skills and digital skills (European Commission, 2021). Therefore, according to many institutions, companies and organisations, the future lies in digitally boosting the world’s economies. But despite this growing demand, there is a shortage of STEM specialists, most acute in engineering and technology sectors, and particularly worrying in terms of gender shortages. In fact, an EC study (2019) states that 53% of companies experienced great difficulty in finding STEM professionals. In terms of gender gap, the EU study related to the widening gender gap in the digital sector (European Commission, 2020a) found that there are three times more men than women with technology and engineering degrees, only 17% of specialists are women and women earn 20% less than men. In fact, it is estimated that an annual productivity loss in the European economy of around 16 billion euros is due to women leaving their STEM jobs. As a result, the EU is launching strategies to increase women’s participation in STEM sectors, focusing on challenging gender stereotypes, promoting STEM skills and education and targeting more women entrepreneurs. Thus, the integration of women is essential in this socio-economic context and active participation in STEM sectors is an indispensable ingredient for a sustainable, fair and equitable digital economy and society. It is therefore crucial to know at what point in school development girls lose interest in STEM fields and the real reasons for this behaviour, as this trend is a social and economic problem due to the heavy waste of human resources.

In this social and economic context, education plays a key role, as there is a strong need to foster motivation in STEM, especially among girls from an early age. This means that teachers and education systems must be prepared for this challenge, which is why gender equality and STEM education and its integration into the educational curriculum are of paramount importance. In fact, there is a large amount of research that has analysed the reasons why girls and young women do not choose to study STEM disciplines (Henriksen et al., 2015; Mainhard et al., 2018; Menacho et al., 2021; Microsoft, 2017; Palmer et al., 2017; Salmi et al., 2016; Stoet & Geary, 2018; Vennix et al., 2018). But in addition, this sharp decrease in STEM vocations among girls, is also occurring in the educational and professional context in Spain, where the figures are increasingly alarming (Mateos-Sillero & Gómez-Hernández, 2019; Ministerio de ciencia e innovación del Gobierno de España, 2021; OECD, 2018).

As a consequence, the main objective of this study is to detect barriers, supports and gender gap in the choice of STEM studies in secondary school students (Compulsory Secondary Education, CSE) in the last two years (3rd and 4th, ages 14–16) and how these differ by gender. But in addition, we will also focus on analysing teachers’ perceptions, beliefs and training on STEM and gender. To achieve these objectives, a set of specific topics will be addressed, taking into account all previous research on STEM and gender among teachers and students (Henriksen et al., 2015; Mainhard et al., 2018; Menacho et al., 2021; Palmer et al., 2017; Salmi et al., 2016; Vennix et al., 2018; Gil-Quintana et al., 2020; Cheng et al., 2020; Dasgupta and Shout, 2014; Ahmed and Mudrey, 2019; Diekman et al., 2010; Olmedo-Torre et al., 2018; Starr, 2018; Bloodhart et al., 2020). Thus, we will focus on questions of gender inequality in relation to their present and past hobbies and tastes, participation in out-of-school STEM activities and whether they are highly motivated and supported by their parents and teachers. But we will also focus on their STEM role models and stereotypes, especially female ones, as well as on their positive and negative attitudes towards STEM disciplines, delving into how they perceive the impact of these disciplines as a social good. These topics are considered because the previous research indicates that they have a strong influence and weight in the decision to pursue STEM studies, especially among girls. Therefore, the specific objectives we are pursuing with this study are:

- To better understand students’ beliefs, attitudes and motivation towards STEM, especially those of girls.
- To analyse what out-of-school activities they are interested in and what hobbies they have, to see if there is a relationship between these issues and their interest in STEM. In addition, consideration will be given to whether there are gender differences on these issues.
- To analyse teachers’ beliefs and training on STEM and gender both in secondary education and in the general context, with a special focus on female students.
- To analyse teachers’ actions to guide students towards STEM, specially among female students.

This paper is organized as follows. Section II describes the methodology and sample. Section III shows teachers’ and students’ attitudes towards STEM. Section IV discusses the results and Section V provides the conclusions of the study.

## Theoretical background

As for research related to the lack of girls in STEM, one of the earliest analyses, dating back to the 1960s, Rossi (1965), already concluded that parents and teachers should motivate girls and make them self-sufficient and independent to develop analytical skills. Thus, much research has emerged since the 1970s to explain the gender gap in science (Shepard & Metzler, 1971) and the concept of gender stereotypes has also been the subject of study since the 1980s by authors such as Eagly & Steffen (1984), who claimed that the female gender was stereotyped with an affective, intuitive, empathetic and passive personality.

More recently, there is a strong tendency to believe in the technological paradox of gender equality, based on the fact that the more egalitarian a country is, the less women study technology and science. Thus, the research presented by Stoet & Geary (2018) looked at almost half a million teenagers in 67 countries, and in more than 60 countries far fewer females study science or technology than males. The research suggests that socio-economic status plays an important role, with less egalitarian countries adding pressure to enter higher-paying professions, often related to STEM sectors. Thus, women are more likely to enter these fields in countries with less social welfare. On the other hand, a study conducted by Microsoft (2017) among 11.500 girls across Europe establishes a connection between female role models and girls’ interest in STEM, so girls who know female role models show almost twice the level of interest in STEM as girls without them. The problem is accentuated by the fact that 64% of the girls interviewed were unable to identify any women who excel in STEM. In addition, the study shows the need to feel supported by their families, with 81% of girls who receive parental support reporting a predisposition to pursue STEM studies. Indeed, the impact of the close environment (family, relatives, teachers) is also corroborated by other studies (Henriksen et al., 2015; Mainhard et al., 2018; Menacho et al., 2021; Palmer et al., 2017; Salmi et al., 2016; Vennix et al., 2018). Besides, the need to make female STEM role models visible, especially in schools, is also demonstrated by other studies (Gil-Quintana et al., 2020; Cheng et al., 2020). Thus, Microsoft’s research proposes to boost girls’ interest in STEM by providing information about the benefits and impact of STEM in people’s lives, which even doubles their interest. In addition, the study underlines the need to provide real life experiences to girls, since only 31% of European girls participate in out-of-school STEM activities. In this way, they state the need to foster a learning environment that promotes curiosity and experimentation. In fact, other studies (Dasgupta and Shout, 2014; Ahmed and Mudrey, 2019) also conclude that girls are more interested in STEM subjects when they are taught from an applied perspective and Diekman et al., (2010) state that girls tend to opt for studies in which they can help others. Furthermore, other research studies support the positive impact of science and technology outreach activities to improve perception of STEM (Henriksen et al., 2015; Palmer et al., 2017). Finally, the study by Olmedo-Torre et al., (2018) claims that girls consider social stereotypes and immediate environment as the main reasons for low female enrolment in STEM. This idea of preconceived and distorted stereotypes in STEM was also analysed in (Starr, 2018; Bloodhart et al., 2020).

On the other hand, taking into account these issues and some of the most important factors identified, education systems and teachers need to be aware of the career opportunities that STEM education opens up (OECD, 2015). It is therefore important to convey a positive and inclusive image of STEM, and education should promote science open to new sources of interest and enjoyment for young people, with a special focus on female students. In fact, Unesco reports that the lack of female representation in STEM sectors is holding back progress towards sustainable development, so there is a need to understand the barriers (Chavatzia, 2017). The report emphasises stimulating their interest from the earliest years, including teacher training in STEM and gender and the development of gender-sensitive curricula, so schools have a key role to play in encouraging girls’ interest in STEM and providing them with equal opportunities to access STEM education. But this issue is also addressed by the 2030 Agenda for Sustainable Development, where the terms STEM, innovation and gender are crucial. The 2030 Agenda places particular emphasis on SDGs 4 and 5 (Sustainable Development Goals) (United Nations, 2021), which speak of quality, inclusive and equitable education, as well as gender equality and the empowerment of girls. The 2030 Agenda also calls for countries to increase their investment in STEM education by ensuring equal access for girls. In fact, in countries with gender-neutral curricula, girls’ motivation towards STEM is higher (Russia, Finland, Sweden, Sweden). For example, in Finland, 62% of female students understand the importance of STEM sectors for their future careers (Microsoft, 2017; OECD, 2016). Moreover, Sweden, one of the countries with the highest proportion of women in STEM, integrates gender training for teachers (World economic forum, 2019) and Croatia will integrate a STEM curriculum into the national school system (Institute for Youth Development and Innovation, 2021). Finally, in countries such as the Netherlands, programmes on gender awareness, gender stereotypes in STEM and gender-sensitive career guidance are taught at secondary school level (Expertise Centrum Gender Diversite it in Bèta, 2020).

Furthermore, this decline in STEM vocations, especially among girls, also happens in Spain, where the figures are also very worrying. In this regard, the latest Spanish government report on women scientists in 2021 (Ministerio de ciencia e innovación del Gobierno de España, 2021) points out that in engineering and architecture studies, one in four graduates is a woman (25%), which shows clear and persistent gender imbalances. Besides, the study shows a lower presence of female researchers in STEM areas, particularly in engineering and technology, less than 13%. Furthermore, women do not participate equally in the system, with only 23% of research institutes headed by women, and they have lower success rates (43%) and receive less funding than their male counterparts although the proportion of female researchers applying to R&D (Research and Development) calls is increasing, Indeed, some studies (Mateos-Sillero & Gómez-Hernández, 2019) show that young women pursuing STEM studies are decreasing. Specifically, the percentage of women in Electrical and Electronic Engineering was 12%, 22% in Industrial Technologies Engineering and Aeronautical Engineering and 20% in Telecommunications Engineering. In addition, the results of the latest PISA report (OECD, 2018) show that in Spain boys scored 6 points higher than girls in mathematics and scientific vocations continue to be predominantly male. However, this gender inequalities in STEM fields are greater in the EU than in Spain. In fact, in the EU, women with a PhD degree in computer science represent 23% and in Spain 26%, while in engineering 29% in the EU and 38% in Spain.

## Methods and experimental

A quantitative descriptive, correlational and explanatory descriptive design (Skinner, 2020) has been carried out with the aim of finding out the motivation of secondary school girls towards STEM studies, understanding why there are currently fewer girls studying these degrees and finding out the teachers’ opinion about this lower participation of girls. This type of statistical analysis allows to generalize the results, to have a greater control over the phenomena.

### The InGenias project

The InGenias project was launched at the Technical School of Telecommunications Engineering of the University of Valladolid (UVa), with the aim of promoting technological vocations among secondary school students, especially girls, making university female students and teachers the main roles in achieving this goal. The project aims to increase these future vocations by showing the more social and human perspective of technology and how it can have a positive impact on the quality of life of citizens and society. In this way, InGenias responds to the needs of a society that is suffering a significant decline in technological vocations, especially in the female sector. The methodology of InGenias is as follows:

1. To establish contact with Secondary Schools to offer the activity. This contact begins in September, to plan the visits from February to April.
2. To train teams of female and male teachers and students attending secondary schools. Experts in scientific dissemination propose communication strategies with a special focus on making the presence of women in STEM more visible.
3. To develop prototypes and scientific-technological experiments of a social and sustainability nature (in parallel with the previous actions).
4. To disseminate talks and experiments/prototypes in Secondary Schools (3rd and 4th CSE). These courses are crucial as students particularly lose interest in Technology and Engineering.
5. To send surveys to secondary teachers and students to try to analyse their perspective and motivation for STEM and compare their views.

### Participants’ characteristics

In this research study, non-probability purposive sampling (Etikan & Bala, 2017) was used with two distinct groups: a group of students and a group of teachers. In the group of students, 1562 students aged between 13 and 18 years (µ = 14.96; σ = 1.018) participated, 49.7% were male and 50.3% female. In the group of teachers, 432 teachers between 24 and 68 years of age participated (µ = 45.54 σ = 9.094). In the group of the teachers, 27.5% were men and 75.5% were women. Furthermore, 69.4% of the teachers have a STEM degree compared to 30.6% who do not have a STEM degree. The years of teaching experience of the teachers ranged from 1 to 40 years (µ = 15.77 σ = 10.4251). The criteria for the selection of the sample were students and teachers from the secondary schools that voluntarily decided to take part in the InGenias Project.

### Data collection and analysis procedure

The questionnaires were designed ad hoc, based on a systematic review of the scientific literature in the framework of the InGenias project on STEM (Dasgupta & Stout, 2014; Fouad et al., 2010; Henriksen et al., 2015; Mainhard et al., 2018; Menacho et al., 2021; Molina-Gaudo et al., 2009; Palmer et al., 2017; Salmi et al., 2016; Vennix et al., 2018) which favored greater precision in the wording of the items. In fact, questions related to the influence and support of teachers and parents/guardians were extracted from (Mainhard et al., 2018; Dasgupta & Stout, 2014; Fouad et al., 2010; Salmi et al., 2016; Menacho et al., 2021). Topics regarding out-of-school activities were taken from (Vennix et al., 2018; Henriksen et al., 2015; Salmi et al., 2016) and issues related to interest, abilities/skills or enjoyment from (Palmer et al., 2017; Dasgupta & Stout, 2014; Molina-Gaudo et al., 2009). Furthermore, the alignment of personal goals and values with STEM disciplines and those related to external motivation were extracted from (Dasgupta & Stout, 2014). Finally, questions related to role models were taken from (Gil-Quintana et al., 2020; Cheng et al., 2020). Thus, two types of questionnaires were designed, one for students and one for teachers. Both were carried out using Google Forms and were answered anonymously.The teachers’ questionnaire included a total of 25 dichotomous response questions (yes or no). On the other hand, the student questionnaire included the next questions:

- 35 dichotomous questions (yes or no).
- 3 LIKERT scale questions (10-point scale):
  - I participate in out-of-school STEM activities.
  - My parents/tutors encourage me to STEM activities.
  - My teachers encourage me to STEM activities.
- 7 open-ended questions:
  - Age.
  - Why do you or don’t you think there are different toys according to gender?
  - Adjectives of your personality which describe yourself.
  - What would you like to do when you grow up?
  - What skills/abilities do you think you need to have to study STEM?
  - Why do you or do you not find people who are professionally engaged in STEM attractive, striking or curious?
  - Mention a famous or well-known STEM role model.

Furthermore, the reliability of the questionnaires was tested with Cronbach’s alpha, a widely used method to determine the reliability of a questionnaire (Amirrudin et al., 2021; Cortina, 1993). Cronbach’s alpha results are considered good if the value is above 0.6 (Cicchetti and Sparow, 1981). In fact, for the students’ questionnaire, carried out on all variables, a good result of 0.714 was obtained. And for the teachers’ questionnaire a reliability of 0.739 was obtained, so it can be stated that the reliability obtained is good for both questionnaires.

All the answers were collected and processed using the SPSS 24.0 statistical package for Windows. In all the tests, a confidence level of 95% was established. Furthermore, frequencies, means and percentages were established for each variable. In addition to the descriptive statistics, the Chi-square test was performed to estimate whether there was an association between two variables. The Cramer’s V or Phi test was also performed to find out the strength between the two variables and the Lambda test to measure the intensity and check whether it was possible to use one variable to predict another. In addition, a complementary qualitative analysis was done using word clouds. A word cloud is defined as a visual representation of words structured according to their frequency (Jayashankar and Sridaran, 2017). In the case of the open-ended questions, a complementary content analysis was carried out using word clouds, which allowed us to get to know some of the students’ answers graphically, as word clouds could be one of the most influential visualisation paradigms for most condition-independent analyses (Snyder, 2019). For this purpose, all open-ended responses provided by the participants were selected and copied into a word cloud tool.

## Results

First of all, we will explain all the results related to the students’ questionnaire, and then we will describe the results of the secondary teachers’ questionnaire.

### Secondary students’ results

The results from secondary school students will be divided into different sections. The first two sections: beliefs, attitudes and motivacion towards STEM and STEM role models are related to the first objective of this study, i.e. to find out about students’ interest in STEM, with a special focus on girls. And the last two sections: tastes, hobbies and personal qualities and engagement in out-of-school STEM activities, are related to the second objective, i.e. to analyse what out-of-school activities they are interested in and what hobbies they have, to see if there is a relationship between these issues and their interest in STEM. In addition, consideration will be given to whether there are gender differences on these issues.

#### Beliefs, attitudes and motivation towards STEM

This section will describe students’ attitudes and motivations towards STEM studies, with a special focus on gender. Table 1 shows the results obtained taking into account both genders, male and female students. Indeed, Table 1 shows that girls (53.8%) were less interested than boys (67.5%) in pursuing STEM-related studies, although more female students wanted to continue their studies (95%) than male students (88.8%). However, this contrasts with the fact that female students found people working in STEM sectors more attractive (71.1%) compared to male students (62%). It was analysed why they did not opt for STEM studies in the future, it was found differences between girls and boys. Ranking the answers from highest to lowest, we could see that the first two answers coincided for both: “because I don’t like it” (girls: 71.5%; boys: 63.1%), “because I don’t see myself capable” (girls: 47.5%; boys: 46.3%). However, the other responses were different according to gender. For boys they were in decreasing order: “I don’t understand what they do” (22.4%), “because it’s geeky” (11%) and “they don’t contribute anything socially” (10.6%). On the contrary, girls scored in decreasing order: “they don’t contribute anything socially” (24.8%), “I don’t understand what they do” (14.6%) and “because it’s geeky” (5.1%). Thus, the biggest difference was found in “don’t contribute anything socially” (boys: 10.6%; girls: 24.8%).

**Table 1:** Descriptive frequency analysis of the dichotomous yes/no questions about actitudes and motivation towards STEM

		Both genders	Boys only	Girls only
		Yes	Yes	Yes
Do you want to continue studying when you finish CSE/High school?		91.9%	88.8%	95%
Would you like what you study to be linked to Technology or Science?		60.7%	67.5%	53.8%
Do you find people who are professionally engaged in Science, Technology or Engineering attractive, appealing or curious?		66.7%	62%	71.1%
Do you consider that you have to have any special skills or abilities to study these careers?		60.4%	56.8%	64%
Students who study STEM need to	Be hard-working	11.2%	12.4%	10.1%
	Have a logical/scientific midset	6%	5.8%	6.2%
	Have skills and abilities	17.7%	14.4%	20.9%
	Have interest	19.4%	15.3%	23.4%
	Be curious	1.94%	0.9%	3.4%
One reason not to choose STEM studies is	Because I don’t see myself capable	46.9%	46.3%	47.5%
	Because I don’t like it	67.3%	63.1%	71.5%
	Because I don’t understand what they do	18.5%	22.4%	14.6%
	Because it’s geeky	8%	11%	5.1%
	Because I don’t think it contributes anything socially	8%	10.6%	5.5%
One of the most relevant reasons for choosing STEM studies is	For social recognition	27.1%	29.5%	24.8%
	For earning a lot of money	45.8%	55.9%	35.9%
	For helping others	54.5%	49.7%	59.2%
	To work in a team	43.4%	43.3%	43.5%
	Because of the employment opportunities offered by these careers	64.2%	66.9%	61.6%
	For self-improvement	55.8%	52.2%	59.3%
	To improve society	46.9%	40.1%	53.6%

Source: own elaboration

From the Table 1 above we can see why students would choose STEM studies. We found from highest to lowest the following reasons for male students: “job opportunities” (66.9%), “earning a lot of money” (55.9%), “self-improvement” (52.2%), “helping others” (49.7%), “working in a team” (43.3%), “improving society” (40.1%) and “social recognition” (29.5%). On the other hand, for female students the reasons were different, as can be seen below: “job opportunities” (61.6%), “self-improvement” (59.3%), “helping others” (59.2%), “improving society” (53.6%), “working in a team” (43.5%), “earning a lot of money” (35.9%) and “social recognition” (24.8%). Thus, the biggest difference according gender was found in “earn a lot of money” (boys: 55.9%, girls: 35.9%). But there were also important differences between boys and girls when it came to choosing STEM studies for the reasons “helping others” (around 10%) and “improving society” (around 13%). Finally, Table 1 shows that 64% of girls and 56.8% of boys thought that it was necessary to have special skills/abilities to study STEM. Thus, if we list the skills most valued by both genders from the highest to the lowest, we had: having interest, having aptitudes for STEM, being hard-working, having a logical/scientific mentality and finally being curious. However, girls generally valued all the qualities more highly than boys, so it could be concluded that girls were more demanding in terms of the skills needed to study STEM. Specifically, the qualities that showed the greatest differences between both genders were: having interest (males: 15.3%, females: 23.4%) and having aptitude and skills for STEM activities (males: 14.4%, females: 20.9%).

Table 2 shows the correlation results (Lambda and Phi correlation) when analysing the reasons why students would choose STEM studies and their gender. Firstly, it should be noted that certain variables had a Chi-square value greater than 0.05 (no correlation), namely: “gender” and “my teachers encourage me to do STEM activities” (Chi-square significance = 0.685), and “gender” and “my mother/father or tutor encourage me to do STEM activities” (Chi-square significance = 0.148). It could be observed, there was a relationship between gender and the choice of STEM studies to “earn a lot of money”, as the Phi correlation value is 0.201 (moderate relationship). This fact was also corroborated in Table 1, as 55.9% of boys thought this reason was important for choosing STEM studies, compared to only 35.9% of girls. Besides, there was a very weak relationship between the rest of the variables and gender (value below 0.200). However, the highest values were observed among the female gender and the responses “helping others” (0.95) and “improving society” (0.135).

**Table 2:** Lambda and Phi correlation in relation to students’ answers

		Lambda		Phi correlation	
		Value	Asymptotic standard error	Value	Asymptotic standard error
V1: Gender V2: one of the reasons to choose STEM studies is to help others.	Symmetrical	0.049	0.031	0.095	0.000
	V1 dependent	0.089	0.033		
	V2 dependent	0.006	0.039		
V1: Gender V2: One of the reasons for choosing STEM studies is to improve society.	Symmetrical	0.103	0.031	0.135	0.000
	V1 dependent	0.129	0.035		
	V2 dependent	0.077	0.037		
V1: Gender V2: One of the reasons for choosing STEM studies is to earn a lot of money.	Symmetrical	0.164	0.030	0.201	0.000
	V1 dependent	0.196	0.031		
	V2 dependent	0.128	0.036		

Source: own elaboration

#### STEM role models

In this section we will describe the STEM role models of students (boys, girls). On the one hand, the closest role models (family, friends) and, on the other hand, famous people. In the next sections, the difference between the two genders will also be analysed. Thus, when secondary school students were asked whether they knew people in their immediate environment who worked in STEM sectors, Table 3 shows that the levels were similar for girls and boys, with slightly higher percentages for girls. However, it was observed that they knew more people of the male gender, as could be seen in particular in the responses: father/mother, uncle/aunt and male/female cousin. In addition, it was important to notice that 19.8% of male students and 22% of female students did not know anyone in their environment who worked in these professional environments.

**Table 3:** Descriptive frequency analysis of the dichotomous yes/no questions about STEM role models

		Both genders	Boys only	Girls only
		Yes	Yes	Yes
I know someone in my close environment who works in STEM:	To my father/guardian	15.4%	14.3%	16.4%
	To my mother/guardian	7%	7.3%	6.6%
	To my uncle/s	17.9%	16.1%	19.6%
	To my aunt/s	10.1%	7%	13.1%
	To my male cousin/s	11.4%	9.5%	13.2%
	To my female cousin/s	8.8%	4.9%	12.7%
	To other family members	19.8%	17.4%	22.1%
	To Friends or acquaintances	26.9%	20.4%	33.3%
	To teachers	26%	21%	30.9%
	I do not know anyone in my environment who works in STEM	19.8%	22%	17.7%

Source: own elaboration

**Fig. 1:**
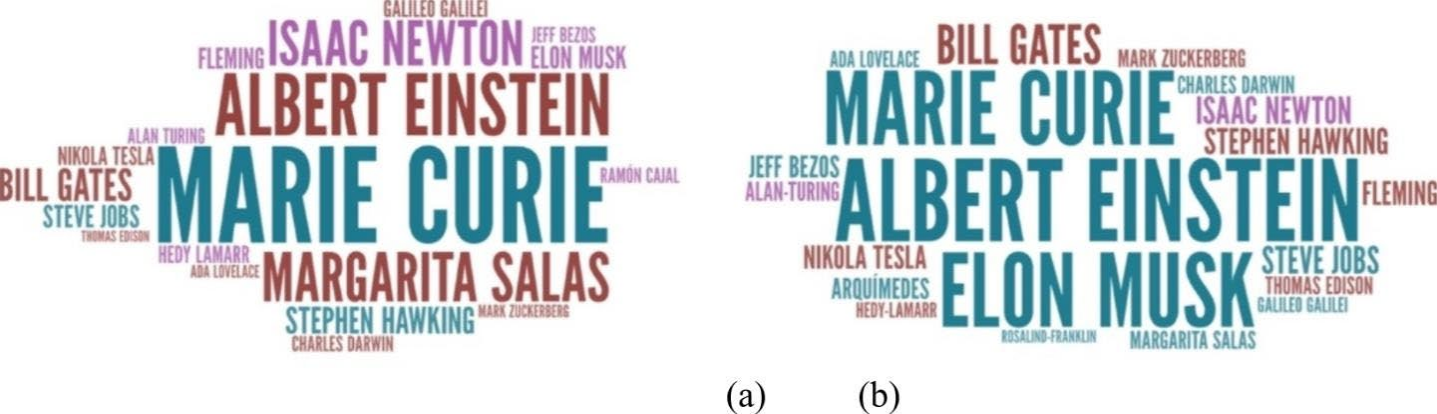
Famous role models that secondary students know (a) girls (b) boys. Source: own elaboration

Furthermore, the word cloud in Fig. 1 shows that the famous STEM role models were similar for both genders with the exception of Margarita Salas and Marie Curie, which were more recurrent for girls, and Albert Einstein, which was more recurrent for boys. With regard to technology, engineering or mathematics role models, in Fig. 1 we only could find Mark Zuckerberg, Steve Jobs, Elon Musk and Jeff Bezos, although to a much lesser extent than the most frequently mentioned (between 75-85% less). But also, the only recognised female STEM role models were Marie Curie and Margarita Salas, and even more, students did not identify women in other STEM areas such as technology, engineering or mathematics. Finally, some students also named science youtubers (hiperactina, Débora science, Nate gentile, Toro Tocho or Dante GTX), but to such a lesser extent that they did not appear in the word cloud.

#### Tastes, hobbies and personal qualities

In this section we will relate students’ tastes and hobbies to their attraction to STEM disciplines according to gender. In addition, their personal qualities will also be described, with an emphasis on gender differences. Table 4 shows the results of the correlation when looking at the toys they played with in their childhood and their likes and dislikes, to see if there was a relationship between these and what they thought about STEM. It was necessary to analyse the association between the variables by performing the Chi-square test, where if the Chi-square value was less than 0.05 there was correlation between the variables. Thus, the pairs of variables in Table 4 had a Chi-square result of less than 0.05. Table 4 shows that although there was a correlation between variables, we could only say in one particular case that we could predict one of the variables by looking at the other, namely between “gender” and “during my childhood I played with dolls” (lambda of 0.544), which means that dolls could be predicted by (female) gender. However, toys did not seem to be a determining factor for their future, as although there was a relationship between “playing with dolls” and liking “writing, language, reading, literature, history, music, art”, this intensity was very weak (0.091); as well as the relationship between “playing with legos, puzzles, cars” and “studying something related to STEM” (0.122).

**Table 4:** Lambda and Phi correlation in relation to students’ answers

		Lambda		Phi correlation	
		Value	Asymptotic standard error	Value	Approximate significance
V1: Gender V2: during my childhoood I played with dolls	Symmetrical	0.490	0.027	0.561	0.000
	V1 Dependent	0.544	0.027		
	V2 Dependent	0.422	0.035		
V1: During my childhood I had games and toys related to construction, legos, puzzles, cars etc. V2: Would you like what you study to be linked to technology or science?	Symmetrical	0.031	0.015	0.122	0.000
	V1 dependent	0.000	0.000		
	V2 dependent	0.037	0.037		
V1: When you were a child you played with dolls V2: Do you like more subjects related to: Writing, Language, Reading, Literature, History, Music, Art… or with Mathematics, Physics, Chemistry, Biology, Technology….	Symmetrical	0.000	0.000	0.091	0.000
	V1 dependent	0.000	0.000		
	V2 dependent	0.000	0.000		

Source: own elaboration

**Fig. 2:**
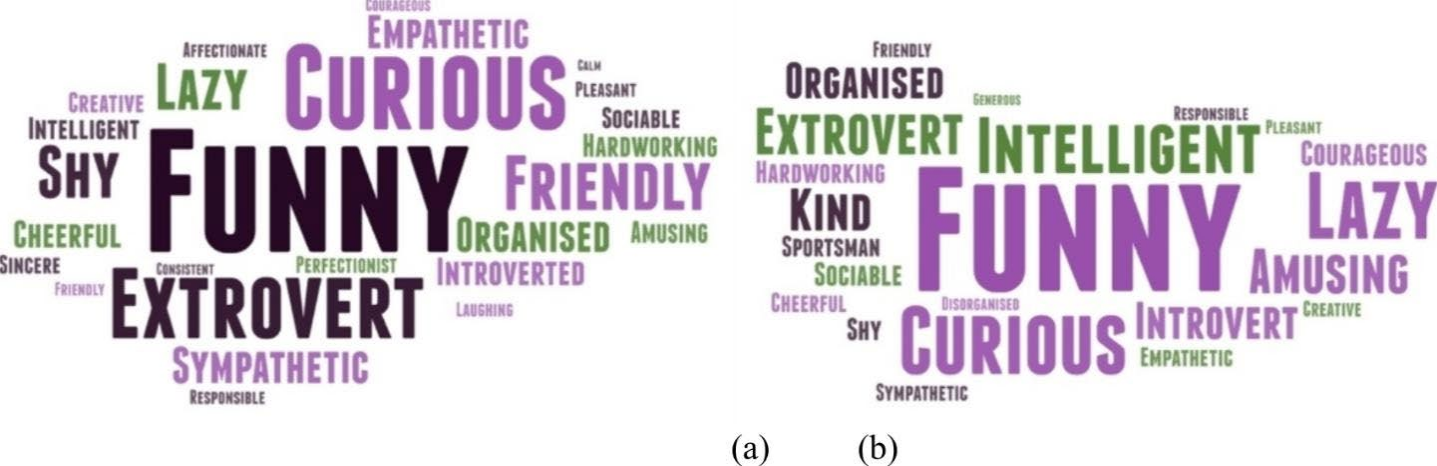
Personal qualities with which secondary school students feel identified (a) girls (b) boys. Source: own elaboration

Moreover, Fig. 2 shows the personal qualities with which girls (a) and boys (b) identified. Both of them perceived themselves as “funny”, “curious” and “extrovert”. However, it was observed that boys did perceive themselves as “intelligent” and “lazy”, while girls also perceived themselves as “lazy” to a lesser extent and the adjective “intelligent” hardly appears. It was also worth noting that girls very often perceived themselves as “empathetic” and “shy” but boys did not. In contrast, boys saw themselves as “courageous”, an adjective that girls rarely used.

**Fig. 3:**
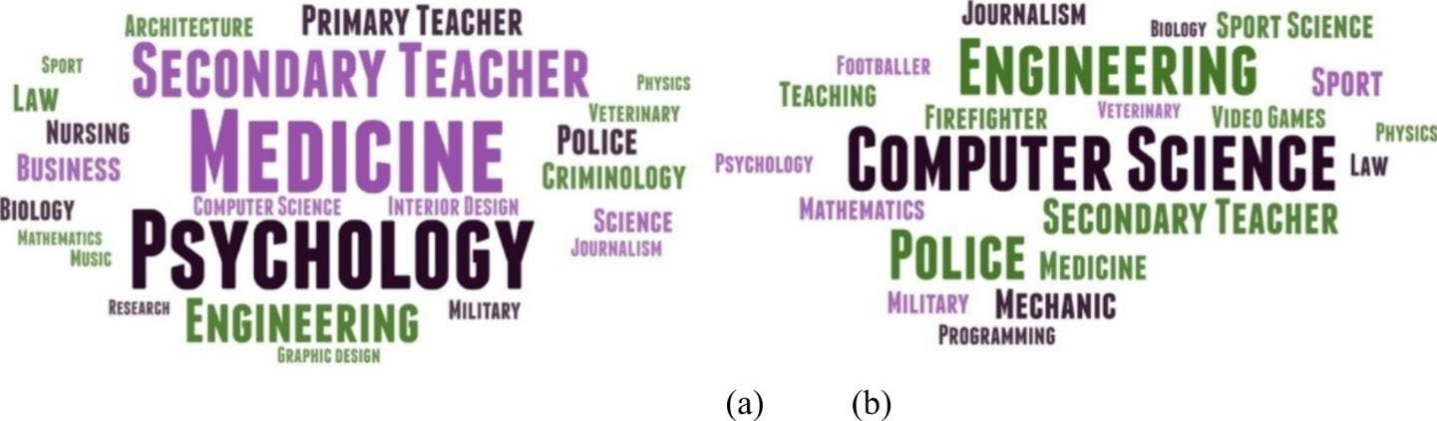
Future careers that young people in secondary education wanted to pursue in the future (a) girls (b) boys. Source: own elaboration

Figure 3 shows what girls (a) and boys (b) wanted to do in their future. It could be seen that girls’ preferences are for Medicine, Secondary School Teacher, Psychology, Primary School Teacher and, to a lesser extent, Engineering. In contrast, boys prefered Engineering, Computer Science, Police or Secondary School Teacher.

#### Engagement in out-of-school STEM activities

This section will explain whether students engage in STEM-related extracurricular activities, whether they were encouraged to do so, and gender differences. The results show that the students’ families encouraged their children to pursue STEM activities in a similar way regardless of gender, although the percentage was slightly lower for girls (males: 45.9%; females: 42.2%; Both genders: 44%). The same was true for teachers, as both girls and boys felt that they encourage them to pursue STEM activities in a similar way (males: 61.7%; females: 62.7%; both genders: 62.2%). Furthermore, Table 5 shows the results to questions related to their participation in out-of-school STEM activities and the level of involvement of teachers/family in such participation (1 being the lowest and 10 the highest). Analysing the results, it could be observed that the encouragement of families and teachers was rather low for both genders, as well as their participation. But in addition, these levels were always lower for girls than for boys.

**Table 5:** Encouragement of teachers and families to participate in out-of-school STEM activities

	Boys		Girls	
	Media	Standard deviation	Media	Standard deviation
Currently, I frequently participate in activities outside of high school hours related to STEM (“1” being very little and “10” being a lot).	3.74	2.783	3.36	2.550
Currently, my parents or guardians encourage me to participate in activities related to STEM (“1” being very little and “10” being a lot).	4.62	2.952	4.57	2.934
Currently, my teachers encourage me to participate in activities related to STEM (“1” being very little and “10” being a lot).	4.55	2.830	4.51	2.735

Source: own elaboration

### Secondary teachers’ results

The second instrument analysed is the teacher survey. With this instrument it has been possible to collect information on the two main objectives of the project: to analyse teachers’ beliefs and training in STEM and gender both in secondary education and in the general context, and to analyse teachers’ actions to guide students towards STEM (especially girls). Therefore, this section has been divided with both objectives in mind.

#### Teachers’ beliefs and training on gender and STEM

Table 6 shows the means of a descriptive frequency analysis of the teachers’ beliefs and training on STEM and gender questions. Firstly, Table 6 shows that non-STEM teachers had received more gender training (43.9%) than STEM teachers (24.7%), and even more non-STEM teachers perceived that they were currently provided more training (38.6%) than STEM teachers (27%). In terms of training on gender equality in STEM, almost half (48%) said that no specific objectives were included in the curriculum, and a high percentage (43.85%) said that they did not include activities in their subjects either. If we separated STEM and non-STEM teachers, the latter perceive that they included objectives to promote equality to a greater extent than STEM teachers (61% vs. 47%). It was also worth noting that non-STEM teachers were more likely to say that equality training was provided (38% vs. 27%).

Furthermore, the ranking of the answers given by teachers to the question of why they thought girls did not opt for STEM studies was quite similar for STEM and non-STEM teachers. If we placed the reasons given by both in decreasing order we found: “traditions and preconceived ideas” (STEM: 35.5%, non-STEM: 40.9%), “low visibility of female roles” (STEM: 23.3%, non-STEM: 30.3%), “lack of knowledge about STEM studies and their applications” (STEM: 16. 3%, non-STEM: 12.1%), “female students have low self-esteem” (STEM: 11.3%, non-STEM: 11.4%) and “future working conditions in STEM sectors” (STEM: 6%, non-STEM: 6.8%). The biggest difference between both of them was found in “low visibility of female roles”, where non-STEM teachers perceive a greater need for visibility. In this sense, almost half of the teachers think that in STEM sectors gender influences professional development (50.9%). The main reasons most frequently chosen by all teachers (STEM, non-STEM) were “existing wrong and/or outdated stereotypes” (40.7%), “motherhood” (37.3%), “women have less credibility” (28.5%) and “there is a gap in working conditions” (25.7%). Besides, non-STEM teachers observed greater difficulties in everything but “motherhood” (STEM: 37.7%, non-STEM: 36.4%) and “working conditions” (STEM: 26%, non-STEM: 25%). Finally, when secondary school teachers were asked about the reasons for this decline of female professionals in STEM sectors, a very high percentage (44%) attributed it to the persistence of prejudices in a traditionally male sector and almost 30% thought that there was pressure on women and their interests. It was also curious that only 38.4% said that future academic education was a personal matter. Furthermore, it was worth noting that a considerable percentage (22.7%) perceived that governments/educational institutions should do more to promote these disciplines. When separating the analysis by teachers (STEM, non-STEM), more non-STEM teachers believed that there was more pressure on young women, but for the rest of the options both levels were similar.

**Table 6:** Descriptive frequency analysis of the questions answered positively about training and beliefs in gender

		All teachers	STEM teachers	Non-STEM teachers
I have received training with a gender perspective in my degree, postgraduate studies or life-long education		30.6%	24.7%	43.9%
Do you think that female students do not study STEM degrees because of:	By traditions and preconceived ideas	37.%	35.7%	40.9%
	There is little visibility of women’s roles	25.5%	23.3%	30.3%
	Lack of knowledge of STEM studies and their applications	15%	16.3%	12.1%
	For the future working conditions studying STEM degrees	6.3%	6%	6.8%
	Female students have low self-esteem and/or negative self-concept	11.3%	11.3%	11.4%
Do you think that being a man or a woman has an influence on scientific and technological professional development		50.9%	51.7%	49.2%
Male or female status influences scientific-technological professional development for one of the following reasons:	There is social discrimination	19.9%	16.3%	28%
	There is a gap in working conditions	25.7%	26%	25%
	Motherhood	37.3%	37.7%	36.4%
	There is increased effort and competitiveness	13.7%	13%	15.2%
	Women have less credibility, they have more to prove	28.5%	26.3%	33.3%
	Existing erroneous and/or outdated stereotypes	40.7%	40.3%	41.7%
Do you think the education curriculum includes objectives to promote gender equality in STEM degree choices?		51.9%	47.7%	61.4%
Is teacher training provided in gender equality in STEM?		30.6%	27%	38.6%
Why are there fewer women working in STEM fields?	Lack of promotion of STEM studies among women by governments and educational institutions	22.7%	21%	26.5%
	Pressure exerted on young women by socially and culturally constructed identities about what a woman is and what her interests are	29.9%	27%	36.4%
	To the persistence of prejudices about the areas where women or men are more capable	44%	44%	43.9%
	That university education is a personal choice and each person chooses what interests them most and in which they have the greatest potential, independently of external cultural pressures	38.4%	38%	39.4%
	I don’t know why there may be fewer women in STEM fields	22.5%	25%	16.7%

Source: own elaboration

#### Teachers’ actions to guide students towards STEM

In this section, we will focus on the results related to the questions on how teachers guide teachers toward STEM, with a special emphasis on gender. Table 7 shows that 56.7% of STEM teachers included activities in their subjects to promote gender equality in the choice of STEM studies. Furthermore, 62.7% encouraged female students in some special way, the most popular being the visibility of women (46%), followed by positive reinforcement and motivation (41.7%), the development of actions within the subjects and/or the school (34%) and, finally, participation in STEM projects/workshops (23%). When this perception was disaggregated between STEM and non-STEM teachers, the differences between the two were very small. Finally, teachers were asked how important they thought it was that the number of women choosing to study STEM subjects has decreased in recent years, with 1 being the minimum and 10 the maximum, and the average was relatively high 7.91 (σ = 2.054).

**Table 7:** Descriptive frequency analysis of the questions answered positively on the orientation to STEM

		All teachers	STEM teachers	Non-STEM teachers
Do you include content and activities in your classroom programming with the aim of promoting gender equality in STEM?		56.3%	56.7%	55.3%
Do you provide any special incentives for female students to participate in STEM-related activities?		61.6%	62.7%	59.1%
How you encourage girls to participate in STEM-related activities:	Participating in STEM projects/workshops	22.5%	23%	21.2%
	Positive reinforcement and motivation	40%	41.7%	36.4%
	Developing actions/activities within the subjects and/or school	33.1%	34%	31.1%
	Visibilising women in STEM fields	45.8%	46%	45.5%

Source: own elaboration

To conclude the data analysis of teachers, Table 8 shows the pair of variables with significant relationship (Chi-square less than 0.05). It could be seen that it was not possible to predict one variable taking into account the other because Lambda was less than 0.200, but there was a relationship between certain variables, namely between teachers who steered students towards STEM studies and those who also did so especially among female students (moderate correlation 0.251). In contrast, it was quite striking that there was no positive correlation between teachers who encouraged female students and ICT teachers, robotics, technology, programming and/or scientific literacy (Chi-square = 0.061) and the same was true for mathematics teachers (Chi-square = 0.076).

**Table 8:** Lambda and Phi correlation on the association between encouraging students to STEM and encouraging girls

		Lambda		Phi correlation	
		Value	Asymptotic standard error	Value	Approximate significance
V1: Do you steer students towards STEM studies? V2: Do you provide any special incentives for female students to participate in STEM-related activitie	Symmetrical	0.060	0.037	0.251	0.000
	V1 Dependent	0.000	0.000		
	V2 Dependent	0.108	0.065		

Source: own elaboration

## Discussion and implications

### Discussion of students

The two student-related objectives of this study were on the one hand to find out more about students’ beliefs, attitudes and motivation towards STEM, especially girls, and on the other hand to know about out-of-school activities and hobbies The first two sections deal with the discussion related to the first objective and the last two sections deal with the second objective.

#### Beliefs, attitudes and motivation towards STEM

One of the objectives of this research is to understand the beliefs and attitudes of students, especially girls, towards STEM studies, since there are fewer women in these fields than men (Hill et al., 2010; Stoet & Geary, 2018). According to the OECD (2019) in all OECD countries only 1% of girls state that they want to work as STEM professionals, compared to 8% of boys. This pattern is observed in our study, where fewer female students would like to study a STEM-related degree, 53% compared to 67.5% of male students. This trend is also confirmed in the professions preferred by both genders (Fig. 3), more related to health and education for girls and more related to engineering and computer science for boys.

When we delved into analyzing why students would choose STEM degrees, we noticed a significant gender difference from the “earn a lot of money” option, despite the fact that some research has shown that salary is one of the reasons why students choose certain studies regardless of gender (Rafanan et al., 2020). Therefore, this gender difference should be further analysed and cross-checked with other personal or motivational variables, as STEM jobs tend to have a better projection and salary. We also found a weak but existing correlation between being a girl and choosing STEM studies for helping others and improving society. This is important because according to Vázquez-Alonso & Manassero-Mas (2009) the fact of being able to help others is one of the characteristics most valued by students when choosing a degree. So this idea should be reinforced by explaining to students how STEM can help others. For example, STEM disciplines can address issues such as energy saving, climate change, pollution reduction, disease prediction with artificial intelligence, help diagnose neuronal/degenerative diseases (image and signal processing), help people with physical diseases (bioengineering, prosthetics) and so on. In fact, according to Diekman et al., (2010) girls tend to opt for studies in which they can help others, and there is a stereotype that with STEM this goal will not be met. Similarly, the study by Olmedo-Torre et al., (2018) explains that there are many stereotypes regarding STEM professions that influence women’s dislike of STEM studies. In this regard, some studies (Dasgupta and Shout, 2014; Ahmed and Mudrey, 2019) conclude that girls are more interested in mathematics and STEM subjects when they are taught from an applied, hands-on perspective. However, outdated and rigid education in STEM subjects limits exploration and creativity (Banchi & Bell, 2008), so it is crucial for girls to foster their curiosity and learning in STEM disciplines through creative thinking and problem solving (Wang & Degol, 2013). Even more and according to UNICEF (2020), gender-responsive STEM education should include transforming teaching based on learning to think and solve real-life problems, thus enabling students to increase their understanding of how things work. This will enable them to design solutions to real-life problems related, for example, to climate change, health, disease or technology applied in society for the common good.

Perhaps underlying these reasons is the fact that secondary students report at high levels that they do not like STEM studies (girls: 71.5%; boys: 63.1%), especially girls, or that they do not see themselves as qualified to study STEM (girls: 47.5%; boys: 46.3%). In fact, these two reasons are the most important students give when we ask them why they would not choose STEM studies. However, girls generally value all the qualities more highly than boys, so it could be concluded that girls are more demanding in terms of the qualities needed to study STEM. Specifically, the qualities that show the greatest difference between the two genders are: having interest (males: 15.3%, females: 23.4%); having aptitude and skills for STEM activities (males: 14.4%, females: 20.9%). The study by Olmedo-Torre et al., (2018) already stated that girls are not attracted to STEM because of social stereotypes and the immediate environment, so perhaps this makes them not feel capable. On the other hand, we’ve noticed that some girls don’t choose STEM because they think people are geeks or weirdos. In this sense, we know that there is a negative effect between gender stereotypes and STEM identity (Starr, 2018), and therefore, as Starr (2018) says, it is necessary to address in school gender stereotypes in STEM and the stereotypes of genius, nerd, geek that exist in STEM. On the other hand, the students in our study state that they do not know what STEM people do (boys: 22.4%, girls: 14.6%), so it is necessary to expand the educational curriculum in this aspect, since in the Spanish Curriculum subjects of Computer Science, Robotics or Technology are optional and are taught in advanced courses such as 3rd and/or 4th of CSE (14–16 years) (BOCYL, 2016). In this sense, according to Microsoft (2017) most girls are attracted to science and STEM between the ages of 11 and 12, but their interest decreases considerably between the ages of 15 and 16, which means that governments, teachers and parents have 4–5 years to nurture technology vocations before they lose interest altogether.

Another possible reason why girls do not opt for STEM could be that in our research they are more likely than boys to believe that it is necessary to have more qualities to study these disciplines, since girls generally value all the qualities more highly than boys. There is a belief that people have to be “geniuses” to pursue a STEM profession, and these ideas are preconceived stereotypes (Starr, 2018). This perception that it is necessary to be very intelligent may be a difficulty for girls, as they describe themselves as intelligent and courageous less often than boys (Fig. 2). In this regard, the study by Bloodhart et al., (2020) describes how girls do not feel they have the qualities for STEM studies. Furthermore, Makarova et (2019) explain in their study that girls perceive chemistry, mathematics and physics male subjects. Thus, and following the PISA report (OECD, 2016), in countries where girls perform lower than boys in mathematics and science, they also have a significantly lower sense of self-confidence in STEM (Cotner et al., 2020). Thus, one of the suggestions of Microsoft (2017) to strengthen girls’ interest in STEM involves promoting their confidence in equality since, girls are more likely to pursue STEM studies when they are confident that men and women will be treated equally in these disciplines. Consequently, it is vital to instil a positive and inclusive image of science and technology, as the strong digitalisation of society makes it coherent for education to focus STEM learning towards new sources of interest and entertainment, thus generating a new and more embedded perspective of these disciplines in our daily lives.

#### STEM role models

Female role models have an important impact on girls, as a way of feeling identified or connected, but yet their responses only mention Marie Curie and Margarita Salas in the area of science. In addition, girls are not able to identify female role models in technology, engineering and mathematics. This is very negative, as it is essential that girls have role models in all areas of STEM and it is quite appealing for them to be taught in school (Gil-Quintana et al., 2020). Neither do they have current referents or other more disruptive ones such as youtubers, but recent studies are showing that youtube channels and other social networks dedicated to the dissemination of science and technology have a positive impact among the young (Vizcaíno-Verdú et al., 2020). In addition to not knowing famous female role models, the fact that girls have few female role models in their nuclear family (mothers, aunts, cousins) is a problem, as studies show that making female STEM roles visible, makes it more likely that they will choose STEM studues (Cheng et al., 2020). Then and according to Habig et al., (2020), it is necessary to provide students with experiences with professionals, scientists or university students in order for them to eventually choose STEM. Besides, the Microsoft research (2017) also concluded that having visible female role models sparks girls’ interest in STEM disciplines and helps them to picture themselves pursuing these fields.

#### Tastes, hobbies and personal qualities

Delving deeper into girls’ tates and hobbies towards STEM studies, we found in our sample a dependency relationship between playing with dolls and being a girl, which was to be expected as there is a tendency for girls to play more with dolls due to social and gender stereotypes (Mayeza, 2018). However, this predilection does not affect the choice of studies, since although we obtained a relationship between playing with dolls and liking subjects related to language, reading, literature, history, art, this relationship is very weak; and the same occurs with playing with legos, puzzles, cars and studying something related to STEM. Thus, it does not appear that girls’ motivation to study STEM depends on their childhood toys. In fact, some studies have shown that one of the main factors is their close environment, i.e. family support, relatives and peers working in STEM areas, as well as the involvement of teachers and parents (Henriksen et al., 2015; Mainhard et al., 2018; Menacho et al., 2021; Palmer et al., 2017; Salmi et al., 2016; Vennix et al., 2018).

#### Engagement in out-of-school STEM activities

This study revealed that the same actions are not taken on boys and girls when it comes to encouraging their participation in out-of-school STEM activities. In fact, the results show that families and teachers encourage more boys than girls. However, the actions of the immediate environment are determinant (Olmedo-Torre et al., 2018) and it is also known that active learning environments can have a positive impact on pursuing STEM studies and that this impact may be greater for female students (Rainey et al., 2019). Indeed, in our study girls explain that they do very few STEM-related activities, rating the frequency with 3.36 (boys: 3.74). They also state that their parents encourage them little to do STEM activities (girls: 4.57, boys: 4.62), as do their teachers (girls: 4.51, boys: 4.55). Furthermore, in our study we found no relationship between gender and whether teachers or families encourage them to pursue STEM activities, which means that they motivate them equally regardless of gender. This is very worrying, as girls should be encouraged much more. This behaviour is also corroborated in Microsoft (2017) which states that encouragement at school and at home plays a key role for girls and only 41% of them were encouraged by their teachers and 38% by their parents. In this way, according to Zeldin et al., (2008), boys tend to be confident in pursuing STEM studies because of their achievements and continued success, but girls rely on relational episodes in their lives to reinforce their success in male-dominated fields, so external encouragement is more important for girls. Therefore, although the level is low for both genders, it is even lower for girls and we know that it is important to provide multiple opportunities for students to engage in STEM activities, so we have to do more in this direction (Habig et al., 2020). In this way, some studies corroborate the strong impact of out-of-school activities (science and technology outreach) in increasing the positive perception of STEM among young people (Henriksen et al., 2015; Palmer et al., 2017). And other studies (Abe & Chikoko, 2020) point out that families, personality and expectations play a key role in deciding their future studies, so it is important that they engage in activities to realise their expectations and learn STEM.

### Discussion of teachers

The two teacher-related objectives pursued in this study were: to analyse teachers’ beliefs and training on STEM and gender both in secondary education and in the general context, with a special focus on female students and to analyse teachers’ actions to guide students towards STEM. Both objectives have been met, as can be seen in the following sections.

#### Teachers’ perception and training on gender and STEM

Our research study has focused on analysing the motivation and perceptions of secondary teachers to encourage students to pursue STEM studies and their perceptions of the STEM gender gap. We know that this type of guidance is important and needs to be strengthened to increase young people’s vision of their future preferences. Firtsly, it can be state that a very low number, only 30.6%, receive training with gender perspective and it is even more remarkable in STEM teachers (24.7%) compared to non-STEM teachers (43.9%). Therefore, gender training in STEM needs to increase, specially among STEM teachers, as countries with the best results in mathematics and science skills following the PISA reports, provide continuous training for their teachers in STEM disciplines (OECD, 2019). In contrast, the OECD’s 2018 International Survey on Teaching and Learning (OECD, 2019) found that only 39% of teachers in the EU felt skilled in using digital technologies (European Commission, 2020b). In this educational landscape, it is necessary to design competency-based teaching strategies that define the 2030 Sustainable Development Goals (specifically, SDG 4 and SDG 5). Indeed, SDG 4 focuses on ensuring inclusive and equitable quality education, and SDG 5 focuses on achieving gender equality and empowering all women and girls (United Nations, 2021). The EU therefore proposes to strengthen computer science education in schools, but to promote inclusive computer science education in order to have an impact on the number of girls taking up STEM studies. Indeed, gender-responsive STEM education includes transforming education to focus on real-life problems that have an impact on society (health, climate change, disease) (UNICEF, 2020). This involves focusing STEM studies on cohesive learning to develop skills and knowledge through real-world applications. Finally, UNESCO’s 2019 Action Plan report (UNESCO, 2019a; UNESCO, 2019b) proposes focusing on teachers and learning content, expanding gender-sensitive pedagogies and resources, and supporting countries to address gender gaps, especially in STEM subjects.

Regarding gender equality in the educational curricula, almost half of teachers (48%) say that no specific objectives are included and a high percentage (43.85%) state that they do not include activities in their subjects either. In fact, it is more worrying that non-STEM teachers do more activities to promote gender equality than STEM teachers, 61% versus 47%. It is also worth noting that non-STEM teachers report receiving more training than STEM teachers (38% versus 27%). This perception is not in line with the UNESCO report on meeting commitments to gender equality in education (UNESCO, 2018) where the government of some countries, including Spain, state that gender equality is integrated in national school curricula. This leads us to conclude that teachers need to receive more training and work on the inclusion of objectives in the curriculum, to promote strategies in schools to transfer STEM equality to their students in an effective way. In this respect, in countries where educational curricula is gender-neutral, such as Russia and Finland, girls’ motivation towards STEM is higher. Thus, in Finland, 62% of female students say they understand the importance of these subjects and are aware of the kind of jobs they could obtain (Microsoft, 2017; OECD, 2016). In addition, Sweden integrates gender-specific training for teachers, resulting in Sweden being one of the countries with the highest proportion of female STEM graduates (World economic forum, 2019).

When teachers are asked why they think female students do not study STEM, a relatively high level (37%) still think it is due to preconceived or outdated ideas and 25% to the lack of visibility of women’s roles. According to the Microsoft study (2017), showing girls positive female role models in STEM is key, as girls who know female role models feel more empowered to participate in STEM activities (61%). This lack of female STEM role models generates distorted preconceptions of STEM professionals, as 30% of girls imagine men when they describe a scientist, engineer or mathematician. Therefore, there is a need to make female STEM roles more visible, but above all to have the ability to connect with young women, not just focus on models from the past. Beside, according to Kricorian et al., (2020), girls think it is important to be mentored in STEM by someone of their own gender, so it would be interesting to promote mentoring strategies between female students and STEM professionals. As for preconceived ideas, an effort must be made at both the educational and social level to break these social moulds, and in part, showing recent STEM referents will help to develop other models with which future generations will feel identified. These data are corroborated by Makarova et al., (2019) that show that secondary school girls perceive STEM subjects (Mathematics, Physics and Chemistry) as more masculine than boys, and this masculine image decreases the likelihood of choosing STEM. It is also noteworthy that in our study 11% of teachers perceive female students to have low self-esteem, a fact verified by the PISA report (OECD, 2019) which concludes that in countries where girls perform worse than boys in mathematics and science, girls perceive low self-esteem in these subjects. Finally, 15% teachers still think that there is a lack of knowledge about STEM studies and their applications. In this sense and according to the Microsoft study (2017), girls are more interested in STEM when they know their potential, as well as their application to real-life situations. In this sense, the more hands-on experiences a girl receives, the greater her interest, but 39% say they do not receive enough experiences. Furthermore, creativity in the classroom is also key to increasing their motivation towards STEM studies. In fact, girls do not identify with STEM because they feel that these disciplines do not allow them to be creative and 91% of girls describe themselves as very creative.

Furthermore, it is crucial to reverse the perception among secondary school teachers that being male or female influences their professional development (50.9%), particularly among STEM teachers (51.7%). Indeed, the most frequently reasons reflected were “existing erroneous and/or outdated stereotypes” (40.7%) and “difficulties associated with motherhood” (37.3%). These perceptions are aligned with the reasons they give as to why there is a decrease in women working in STEM, since 44% think that it is due to the “prejudices in a traditionally male sector” and nearly 30% due “pressure on women and their interests”. As indicated above, on the one hand, it is necessary to abolish the masculine vision of STEM subjects and transform it with a more open and inclusive vision towards women. On the other hand, STEM should be oriented towards a more practical and social approach that responds to the real challenges of our society, such as climate change, sustainability or diseases. The aim of this change is to break preconceived ideas and recruit female talent by motivating their interest, as it has been shown that young women are creative and prefer to develop practical projects based on real problems. On the other hand, problems associated with motherhood is a persistent reality also corroborated by other current studies (Thébaud & Taylor, 2021).

In addition, it is worth noting that an important percentage (22.7%) perceive that governments/educational institutions should work more on promoting STEM. Thus, many European countries emphasise in-service teacher training, through funding to develop teacher competences and innovative STEM methodologies (European Schoolnet, 2018). Indeed, Finland and Norway receive government funding for STEM development programmes for teachers or the Netherlands has a STEM teacher academy in cooperation with industry (European Schoolnet, 2018). Finally, It is also curious that only 38.4% say that academic future is a personal decision, when this percentage should be much higher in today’s society.

#### Teachers’ actions to guide students towards STEM

Regarding the second objective, related to teachers’ actions to orientate towards STEM, our study reveals that only 62.7% of teachers encourage female students to pursue STEM studies. Moreover, the results have shown a moderate correlation between two variables, so that teachers who guide students towards STEM also encourage female students in a special way. However, it is of concern that there is no positive correlation between teachers of STEM subjects (ICT, robotics, technology, programming, mathematics) and whether they especially encourage female students. Our results indicate that active teaching environments may have a positive impact on their desire to study STEM and this impact may be greater for under-represented students (in this case girls). In fact, only 23% of teachers participate in STEM projects and only 34% develop activities within their subjects and/or school. Therefore, it is crucial to raise teachers’ awareness of this situation and to increase actions to encourage female students to pursue STEM studies. Thus, a close collaboration with the university can generate a positive impact, as some studies have shown (Henriksen et al., 2015; Vennix et al., 2018), a large part of STEM research is generated in university environments, providing them with a great knowledge of job opportunities and future developments. In fact, according to the Microsoft study (2017), when teachers actively encourage girls about STEM subjects, girls are more attracted to these studies and 56% of girls say they would like to have more motivation from their teachers.

## Limitations of the study

This research has focused on detecting barriers, supports and the gender gap in the choice of STEM studies in secondary education, taking into account the perceptions and motivation of students and teachers at this educational stage. However, we have identified some limitations in the study and propose some strategies for future work to address them. One of the limitations of the study that we intend to strengthen is to increase the number of samples of both teachers and secondary school students, in order to have more comprehensive results that provide more reliable conclusions. Another limitation we would like to explore is the integration and analysis of primary school teachers with respect to gender and STEM issues, to have more general results in a wider range of educational stages that would allow us to propose more global strategies from earlier educational stages. Finally, one line of future research is to continue the study by considering the perception of the student guidance departments in secondary schools, since they have to be aware of this decrease in technological vocations and the gender gap in these sectors.

## Conclusion

Motivation for STEM studies has been found to be different for boys and girls. It is more important for boys to earn money and for girls to help others and improve society, so it is important to explain to female students how they can help others and improve society through STEM professions. It has also been analyzed that toys do not affect their future academic decisions. On the other hand, it has been observed that the close environment (families, teachers) encourages boys more than girls or to an equal extent, but studies show that it is more important to encourage girls because boys are more confident that they will perform better. Thus, girls can be encouraged in different ways, such as making STEM subjects more attractive and doing creative, experimental and situation-based projects, to make it more attractive and increase their self-esteem. It is also necessary to build their confidence and skills towards STEM studies and not to think that it will be too difficult for them. In addition, it is also necessary to provide training on STEM applications in the real world and to showcase younger and more contemporary female role models, as well as to integrate new channels of scientific-technological dissemination such as youtubers or influencers. This will make girls feel more connected and identified with these new female role modes, thus eliminating the distorted thinking that STEM professions are geeky.

On the other hand, STEM teachers need to be trained more on gender to increase equitable and inclusive STEM education and performance. A large part of the teachers think that female students do not pursue STEM because of preconceived ideas or a lack of female roles, so the need to show current female role models that young women can identify with is once again latent. In this way, it would be very positive to promote mentoring strategies between female students and STEM professionals. Moreover, secondary school teachers should work on making STEM subjects (Mathematics, Physics and Chemistry) more inclusive and less masculine to attract female talent. It is also noted that a low level of teachers think that girls have a worse low self-esteem, but in reality this is one of the reasons why they do not choose STEM studies, so teachers need to be made aware of this. Besides, there is still a very high level of teachers who do not encourage STEM (especially girls), so more work should be done in this area, as we know that teachers who do encourage in general tend to encourage girls in particular. In addition to change at the educational level, we also believe that institutions and governments need to do more to promote STEM choices among girls, as teachers perceive a lack of support at the STEM level.

Thus, closing gender gaps in STEM education would have a positive impact on employment. In fact, the study (European Institute For Gender Equality, 2021) shows that reducing the gender gap in STEM education areas could help reduce bottlenecks in the labour market, increase the employment and productivity of women and reduce occupational segregation. Even more, the pandemic Covid-19 crisis has put us in a situation where digital technologies become indispensable for the delivery of education, but also exposed the shortcomings of successfully integrating digital technologies into education.

## Data Availability

The datasets used and/or analysed during the current study are available from the corresponding author on reasonable request.
